# Relationship between Altered Platelet Morphological Parameters and Retinopathy in Patients with Type 2 Diabetes Mellitus

**DOI:** 10.1155/2016/9213623

**Published:** 2016-04-12

**Authors:** Tolga Yilmaz, Ahu Yilmaz

**Affiliations:** ^1^Department of Ophthalmology, Beyoglu Eye Research and Education Hospital, Bereketzade Cami Sokak No. 2, Beyoglu, 34420 Istanbul, Turkey; ^2^Department of Ophthalmology, Bagcilar Education and Research Hospital, Mimar Sinan Caddesi, 6. Sokak, Bagcilar, 34100 Istanbul, Turkey

## Abstract

*Purpose*. To investigate whether platelet morphology or function is altered in patients with diabetic retinopathy (DR).* Methods*. This prospective study enrolled 85 healthy controls (HCs) (group 1) and 262 patients with Type 2 diabetes mellitus (T2DM). Patients were subclassified into three groups according to ocular findings: no DR (group 2; *n* = 88); nonproliferative DR (group 3; *n* = 88), and proliferative DR (group 4; *n* = 86). Mean platelet volume (MPV), platelet distribution width (PDW), platelet large cell ratio (PLCR), plateletcrit (PCT) values, and platelet count were measured in the studied groups.* Results*. MPV, PDW, and PLCR levels were significantly altered in groups 2–4 compared with HCs (*p* < 0.05, *p* < 0.05, *p* < 0.05). Compared with group 2, both DR groups had higher MPV and PDW levels, with a significant difference between groups 2 and 4 for both MPV (*p* = 0.036) and PDW (*p* = 0.006). PLCR correlated with retinopathy stage, but no significant difference was found between the DR groups. Platelet count and PCT values were not significantly different between the groups (*p* > 0.05).* Conclusion*. Our findings suggest an association between mean platelet indices (MPI) (i.e., MPV, PDW, and PLCR) and DR stage. Therefore, MPI could be a beneficial prognostic marker of DR in patients with T2DM.

## 1. Introduction

Type 2 diabetes mellitus (T2DM) is an endocrine disease characterized by impaired insulin excretion by the pancreas and insulin resistance of body tissues [1]. Chronic hyperglycemia leads to micro- and macrovascular complications in patients with T2DM; diabetic retinopathy (DR) is the most common and the specific microangiopathy [2]. Diabetes duration, dyslipidemia, genetic factors, obesity, hypertension, smoking, proteinuria, and hypermetropic refractive changes may all play a role in development of DR development [3]. Abnormal insulin activation in patients with T2DM may increase platelet activation and precipitate microvascular complications [4]. Some authors have emphasized the importance of platelet dysfunction in macrovascular (cardiovascular disease [CVD], stroke, and peripheral artery disease [PAD]) and microvascular (nephropathy, neuropathy, and retinopathy) complications, which lead to increased morbidity and mortality in T2DM [4, 5]. Various parameters reflect the condition of platelets, including platelet count, plateletcrit, and mean platelet indices (MPI) (mean platelet volume [MPV], platelet distribution width [PDW], and platelet large cell ratio [PLCR]).

MPV reflects the average size of platelets [6]. It is a marker that indicates subclinical platelet activation and may be increased in some vascular conditions such as myocardial infarction (MI) [6], coronary artery disease (CAD) [7], cerebral ischemia [8], and PAD [7]. Other platelet markers such as PDW, PLCR, and plateletcrit (PCT), which reflect platelet morphology, are also important in vascular events such as atherosclerosis and thrombosis [9]. PDW gives an indication of the distribution of platelet size [10], PLCR indicates the ratio of younger platelet group that has the largest volume [11], and PCT gives the total mass of platelets [9].

Studies have been published on the relationship between DR stages and MPV, PDW, and PCT values, which give information about platelet morphology and function alteration [12, 13]. However, to our knowledge, this is the first study investigating all of the morphologic parameters that reflect subclinical platelet activity (MPV, PDW, PLCR, PCT, and platelet count) between DR stages.

## 2. Materials and Methods

The clinical research was performed between March 2015 and January 2016 at the Department of Ophthalmology, Kocaeli Derince Research and Training Hospital. Patients who were diagnosed as any type of DR in Department of Ophthalmology and diabetic patients who were referred from the Department of Internal Medicine were enrolled in this study. The HC group consisted of healthy individuals with no history of systemic or ocular diseases, who had undergone routine ophthalmic examination. All participants underwent full ophthalmologic examination, and retinopathy status was assessed by fundus photography, fluorescein angiography, and optical coherence tomography. Retinopathy was graded using the International Clinical DR Disease Severity Scale [14].

All of the study procedures were conducted in accordance with the Declaration of Helsinki, and informed consent was obtained from all of the participants after approval from the Institutional Review Board. The study protocol was approved by the Ethics Committee of Kocaeli University School of Medicine.

The present study had a prospective case-control design. Sample size equation for continuous variables of Dell et al., was applied [15]. According to this formula, (*n* = 1 + 2*C*(*S*/*D*)^2^) where *n* was the sample size, *C* was the equation constant with 0.8 power and two sided *α* = 0.05, *S* was the standard deviation of platelet parameters (MPV, PDW, PLCR, platelet count, and PCT) in people, and *D* was the effect size [16]. 72 was the sample size of a single group. Therefore, we designed a 1 : 1 case-control study with groups of 85. We prospectively evaluated platelet parameters (MPV, PDW, PLCR, PCT, and platelet count) in complete blood sample in 262 (138 females, 124 males) patients with T2DM and 85 (46 females, 39 males) age- and sex-matched adult healthy controls (HCs) (group 1). Patients with T2DM were separated into three groups based on the findings of the clinical ocular examination: 88 with no DR (NDR) (group 2), 88 with nonproliferative DR (NPDR) (group 3), and 86 with proliferative DR (PDR) (group 4).

Exclusion criteria were presence of uncontrolled hypertension, anemia (hematocrit below 38%), any cardiovascular disease, stroke, or chronic renal failure; treatment with anticoagulant; or history of alcohol consumption.

Complete blood count samples were taken from each participant and drawn into vacutainer tubes containing 0.04 mL of the 7.5% K3 salt of EDTA and were analyzed within 90 minutes after sampling with a commercially available analyzer (Sysmex 1800 I Automated Cell Counter, Japan).

### 2.1. Statistical Analysis

All values were given as mean ± SD. Differences between the four groups for platelet parameters were evaluated using one-way ANOVA, where applicable. Bonferroni test was used as* post hoc* test after one-way ANOVA. The level of significance was set at *p* = 0.05. All statistical analyses were performed using SPSS for Windows 18.0 software (SPSS Inc., Chicago, IL, USA). Logistic regression analysis was used to assess the associations between platelet parameters and DR stage.

## 3. Results

The average age of the patients was 63.39 ± 8.52 years in group 1, 60.87 ± 8.31 in group 2, 62.72 ± 7.19 in group 3, and 61.66 ± 7.91 in group 4. There was no difference in age or sex of the patients between the four groups (*p* > 0.05) (Table 1).

MPV was 7.42 ± 0.68 fL in group 1, 7.84 ± 0.76 fL in group 2, 7.90 ± 0.85 fL in group 3, and 8.31 ± 0.76 fL in group 4 (Table 1). The blood samples showed a marked elevation in MPV levels in the groups with T2DM compared with controls: group 2, *p* = 0.036; group 3, *p* = 0.016; and group 4, *p* < 0.01. Patients with DR had higher MPV levels among DR stages, but the difference was significant only between group 2 and group 4 (*p* = 0.036).

Mean PDW values were 12.19 ± 1.36% in group 1, 13.02 ± 1.29% in group 2, 13.49 ± 1.18% in group 3, and 13.77 ± 1.26% in group 4 (Table 1). There was a significant difference in PDW levels between the diabetic groups and HCs (group 2, *p* = 0.003; group 3, *p* < 0.001; and group 4 *p* < 0.001). Patients with DR had higher PDW levels among DR stages, but the difference was significant only between group 2 and group 4 (*p* = 0.006).

Mean PLCR values were 28.59 ± 2.28% in group 1, 30.45 ± 2.19% in group 2, 31.31 ± 2.15% in group 3, and 31.71 ± 2.16% in group 4 (Table 1). There was a significant difference in PLCR levels between the diabetic groups and HCs (group 2, *p* = 0.019; group 3, *p* < 0.001; and group 4, *p* < 0.001). The DR groups had higher PLCR levels among DR stages, but the difference was not statistically significant (*p* = 0.993 between groups 2 and 3, *p* = 0.264 between groups 2 and 4, and *p* = 0.833 between groups 3 and 4).

Mean PCT values were 0.27 ± 0.05 in group 1, 0.26 ± 0.06 in group 2, 0.25 ± 0.04 in group 3, and 0.24 ± 0.03 in group 4 (Table 1). There was no statistically significant difference in PCT values between groups (*p* < 0.05). Mean platelet count was 253.76 ± 50.87 10^3^/*μ*L in group 1, 253.86 ± 60.87 10^3^/*μ*L in group 2, 254.77 ± 72.87 10^3^/*μ*L in group 3, and 264.96 ± 64.44 10^3^/*μ*L in group 4. There was no statistically significant difference in platelet counts between groups (*p* < 0.05).

According to logistic regression analysis, there was a 0.91-fold increase in the risk of retinopathy development (OR: 0.913; *p* = 0.07) and a 1.14-fold increase in the risk of proliferative DR (OR: 1.148; *p* = 0.06) as the MPV value increased. There was a 3.10-fold increase in the risk of retinopathy development (OR: 3.106; *p* = 0.002) and a 1.90-fold increase in the risk of proliferative DR (OR: 1.908; *p* = 0.005) as the PDW value increased. There was a 0.67-fold increase in the risk of retinopathy development (OR: 0.676; *p* = 0.07) and a 0.64-fold increase in the risk of proliferative DR (OR: 0.645; *p* = 0.06) as the PLCR value increased.

## 4. Discussion

DM is a multisystemic disease that affects the eyes, kidneys, and peripheral nerves and leads to micro- and macroangiopathy through chronic hyperglycemia [1]. DR is the main cause of retinal vascular disease, which causes blindness between the third and sixth decades of life [2]. Studies have revealed the relationship between increased platelet aggregation and vascular complications of DM [17, 18]. The main problem in diabetic platelets is hypersensitivity against the excretions, causing their activation [19]. Platelets obtained from patients with diabetes secrete stimulants that increase adhesion [20]. Platelet aggregates were seen in the retinal capillaries of diabetic rats in some histological studies [21, 22].

High MPV levels indicate the presence of many large platelets, which are newer, denser, and more active. Therefore, higher MPV values increase the likelihood of vascular complications [23]. CAD [24], OAD [7], and cerebral ischemia [8] were all found to be associated with elevated MPV in previous studies. Ateş et al. reported that MPV values were significantly higher in patients with T2DM compared with controls, whereas there was no significant difference between patients who had background retinopathy and those who developed retinopathy later [23]. Citirik et al. also reported similar results [12]. Ayhan Tuzcu et al. found a correlation between MPV values and DR stage in their study with 192 individuals [25]. Zhong et al. stated that MPV was significantly higher in patients with proliferative DR and proposed that MPV is a risk factor for retinal neovascularization [26]. By contrast, Aydinli et al. advocated that there was no association between MPV and vascular complications in T2DM [27]. In the current study, we found a statistically significant difference in MPV values between patients with T2DM and HCs. When we compared the different diabetic patient groups, we detected a significant difference between patients with PDR and without DR.

PDW is also a specific marker of platelet activation and increases in heterogeneity of platelet volume distribution. Vagdatli et al. reported that MPV and PDW were elevated together in platelet activation but emphasized that PDW is a more specific marker [10]. In the study of Rechcński et al., PDW was found to be an independent risk factor for cardiac mortality and for the occurrence of either death, recurrent MI, or need for another revascularization procedure [28]. Jindal et al. found that PDW was significantly increased in patients with T2DM and reported that it was higher in patients who developed microvascular complications [13]. Citirik et al. found higher PDW levels in patients with diabetes compared with HCs, but this was not statistically significant [12]. In our study, PDW values were significantly higher in patients with T2DM compared with controls. PDW values of diabetic patient groups increased according to retinopathy stage; there was a significant increase in patients who developed PDR compared with patients who had no DR. Logistic regression analysis was performed to assess the effect of PDW values on the risk of developing DR, showing a 3.10-fold increase in the risk of retinopathy development with increased PDW values (OR: 3.106; *p* = 0.002) and a 1.90-fold increase in the risk of proliferative DR as PDW value increased (OR: 1.908; *p* = 0.005).

PLCR is another marker related to platelet volume, and it is an indicator of the largest platelet fraction. An increase in PLCR usually occurs together with an increase in the number of newly produced platelets, which are the largest platelet type. PLCR is usually correlated with MPV, but it is more sensitive to the increase in platelet size. Babu and Basu showed that PLCR is inversely proportional to platelet count and directly related to MPV and PDW [11]. An increase in PLCR may indicate the presence of platelet aggregates, microerythrocytes, and giant platelets. PLCR can serve as useful prognostic factors for long-term mortality in patients after acute MI. Rechcński et al. advocated that PDW and PLCR are prognostic factors after MI and suggested that they could be better than other markers, particularly MPV [28]. Malachowska et al. reported that PLCR was significantly increased in Type 1 DM [29]. The interrelationships between PLCR and T2DM microvascular complications were previously studied by Jindal et al., who found that diabetic patients in general, and patients with diabetes and microvascular complications in particular, have higher values of PLCR but the difference was not significant [13]. More research into this marker is required. In our study, PLCR values were statistically significantly higher in the diabetic groups compared with the HC group. PLCR levels also correlated with the degree of retinopathy, but these differences were not statistically significant.

PCT is defined as the number of circulating platelets per volume of blood. It is a quantitative test for abnormalities in platelet count and calculated as platelet count ×MPV/10^7^ [28]. Demirtas et al. found a significant increase in PCT values in patients with T2DM compared with their control group; however, they did not report a difference between patients with T2DM with or without microvascular complications [30]. Citirik et al. showed that there was no significant difference in PCT level between their diabetic groups and healthy individuals, and there was also no significant difference in mean PCT values between DR stages [12]. Similar conflicting results also exist between platelet count and T2DM; while some studies report no relationship [25, 31, 32], others report a positive correlation [33, 34]. In our study, no significant difference in PCT values or platelet count between the diabetic and HC groups was detected.

Some publications have suggested that proliferative retinopathy is associated with CAD events; therefore, retinopathy and CAD may have similar pathophysiological backgrounds [35, 36]. Platelet markers may have the most important situation in this common pathogenesis. Platelet parameters provide information on the severity of DR and they may also contain tips which are related to the CAD. MPV is also referred to frequently in the pathogenesis, treatment, and prognosis of CVD and also appears to be a useful marker in other systemic and vascular diseases [7, 37]. The role of PDW in systemic disease and specifically in patients with CVD and acute coronary events is yet to be explored. According to some publications PDW is becoming a more valuable marker compared with other platelet markers [10, 26]. Similarly, in our study PDW was found to be the highest independent risk factor compared with the other platelet parameters. There is limited information on the clinical importance of PLCR. Because this parameter is generated by only a few machines (with the Sysmex analyzer being one), few studies are available about the importance of PLCR in systemic and vascular diseases. Studies with larger patient groups are required to clarify its function in patients with T2DM and its role in potential vascular complications. All these indices can be measured by an inexpensive and readily available routine blood count. MPI values may be important in diagnosing and treating patients with DR, and it is possible that higher MPI values will be correlated with the rates of cardiovascular disease and cardiac mortality in these patients. Our study supports the fact that platelet function (MPI) is altered even in patients with DR potentially contributing to increased cardiovascular risk later on.

## 5. Conclusion

In our study, MPI (MPV, PDW, and PLCR) values were significantly higher in patients with T2DM compared with healthy individuals. MPI increases were correlated with the DR stages. In particular, MPV and PDW values were statistically significantly higher in patients who developed proliferative DR compared with patients without retinopathy. A statistically significant difference in PCT and platelet was not observed between the diabetic and HC groups. Based on our results, an increase in MPI values reflects an increased risk for PDR stage.

## Figures and Tables

**Table 1:** Demographic data and comparison of platelet parameters (mean ± SD) for the four study groups.

	Group 1 (control)	Group 2 (no DR)	Group 3 (NDR)	Group 4 (PDR)	*p* value
Age	63.39 ± 8.52	60.87 ± 8.31	62.72 ± 7.19	61.66 ± 7.91	>0.05
Sex	48/37	49/40	48/40	49/38	>0.05
MPV	7.42 ± 0.68	7.84 ± 0.76	7.90 ± 0.85	8.31 ± 0.76	0.036^a^
PDW	12.19 ± 1.36	13.02 ± 1.29	13.49 ± 1.18	13.77 ± 1.26	0.006^a^
PLCR	28.59 ± 2.28	30.45 ± 2.19	31.31 ± 2.15	31.71 ± 2.16	>0.05
PCT	0.27 ± 0.05	0.26 ± 0.06	0.25 ± 0.04	0.24 ± 0.03	>0.05
Platelet count	253.76 ± 50.87	253.86 ± 60.87	254.77 ± 72.87	264.96 ± 64.44	>0.05

DR: diabetic retinopathy; MPV: mean platelet volume; NDR: nonprogressive diabetic retinopathy; PCT: plateletcrit; PDR: progressive diabetic retinopathy; PDW: platelet distribution width; PLCR: platelet large cell ratio.

^a^Between groups 2 and 4.
